# Harm reduction calls to action from young people who use drugs on the streets of Vancouver and Lisbon

**DOI:** 10.1186/s12954-022-00607-7

**Published:** 2022-05-04

**Authors:** Joana Canêdo, Kali-olt Sedgemore, Kelly Ebbert, Haleigh Anderson, Rainbow Dykeman, Katey Kincaid, Claudia Dias, Diana Silva, Youth Health Advisory Council, Reith Charlesworth, Rod Knight, Danya Fast

**Affiliations:** 1 GAT (Grupo de Ativistas Em Tratamentos), Avenida Paris, 4 - 1º direito, 1000-228 Lisbon, Portugal; 2 MANAS, Lisbon, Portugal; 3 ‘Namgis First Nation, Alert Bay, Canada; 4 Coalition of Peers Dismantling the Drug War, Vancouver, Canada; 5 British Columbia Center on Substance Use, 400-1045 Howe Street, Vancouver, V6Z 2A9 Canada; 6 Youth RISE, Sneem, Ireland; 7 Department of Medicine, University of British Columbia, Vancouver, Canada

**Keywords:** Young people who use drugs, Homelessness, Harm reduction, Activism

## Abstract

Vancouver, Canada, and Lisbon, Portugal, are both celebrated for their world-leading harm reduction policies and programs and regarded as models for other cities contending with the effects of increasing levels of drug use in the context of growing urban poverty. However, we challenge the notion that internationally celebrated places like Lisbon and Vancouver are meeting the harm reduction needs of young people who use drugs (YPWUD; referring here to individuals between the ages of 14 and 29). In particular, the needs of YPWUD in the context of unstable housing, homelessness, and ongoing poverty—a context which we summarize here as “street involvement”—are not being adequately met. We are a group of community and academic researchers and activists working in Vancouver, Lisbon, and Pittsburgh. Most of us identify as YPWUD and have lived and living experience with the issues described in this comment. We make several calls to action to support the harm reduction needs of YPWUD in the context of street involvement in and beyond our settings.

## Introduction

Harm reduction is now recognized globally as a cornerstone of addressing drug-related harms [1]. Vancouver, Canada, and Lisbon, Portugal, are both celebrated for their world-leading harm reduction policies and programs and regarded as models for other cities contending with the effects of increasing levels of drug use in the context of growing urban poverty.

In September 2003, North America’s first supervised injection site (known as Insite) was opened in Vancouver’s Downtown Eastside neighborhood [2]. Harm reduction has long been officially prioritized as one of the “four pillars” of the city’s approach to addressing drug-related harms (along with law enforcement, treatment, and prevention), including the current North American overdose crisis driven by the proliferation of illicitly-manufactured fentanyl and related analogues in criminalized drug markets [3]. In addition to Insite, downtown Vancouver is now home to several overdose prevention sites in shelter, housing, hospital, and outdoor environments [4, 5]. Sterile drug use paraphernalia and take-home naloxone overdose antidote kits are widely distributed [6], and two rapid access clinics provide low-barrier access to medication-assisted treatment, including opioid agonist therapy (OAT; e.g., methadone, buprenorphine-naloxone), injectable OAT (titrated daily witnessed injected doses of diacetylmorphine or hydromorphone), and prescription opioids (e.g., hydromorphone tablets) and stimulants (e.g., dextroamphetamine tablets) [7, 8]. The latter are “risk mitigation” prescriptions that were introduced during the COVID-19 pandemic to allow people who use drugs to better self-isolate, and avoid withdrawal and overdose while doing so [9]. Drug checking services are also increasingly available in Vancouver [10].

Portugal decriminalized the use, acquisition, and possession of all drugs in 2000, moving towards a public health approach that integrated harm reduction, prevention, dissuasion, treatment, and reintegration [11, 12]. In Vancouver, there have been growing calls for the decriminalization of substance use, including recent protests during which tested, unadulterated heroin, crystal methamphetamine (meth), and cocaine were distributed to people who use drugs [13–15]. In Lisbon as in Vancouver, there have been concerted efforts to draw people who use drugs into a continuum of care that ranges from the provision of sterile drug use paraphernalia to OAT (primarily methadone in this context) to abstinence-based residential drug treatment. In Lisbon, harm reduction programs are delivered via mobile units and outreach teams and in community health and drop-in centers and shelters [16]. The first mobile drug consumption room was opened in Lisbon in 2019. In 2021, twenty years after the start of decriminalization, the first safer smoking and injection sites were opened in Portugal. While drug checking services have been available at music festivals for some time in this setting [17], they were made more widely available through a twice weekly drop-in program and mobile unit in Lisbon in 2019 and 2022, respectively.

In Vancouver and Lisbon, drug user activists and their allies have waged fierce battles for these policy reforms. There is no question that hard-won interventions like syringe exchanges, low-barrier OAT programs, peer-to-peer overdose prevention (naloxone) programs, and drug consumption sites save lives. However, we challenge the notion that internationally celebrated places like Lisbon and Vancouver are meeting the harm reduction needs of young people who use drugs (YPWUD; referring here to individuals between the ages of 14 and 29).^1^ In particular, the needs of YPWUD in the context of unstable housing, homelessness, and ongoing poverty—a context which we summarize here as “street involvement”^2^ for the sake of brevity—are not being adequately met.

We are a group of community and academic researchers and activists working in Vancouver, Lisbon, and Pittsburgh.^3^ Most of us identify as YPWUD and have lived and living experience with the issues described in this comment. Several of us are playing an active role in shaping harm reduction in Vancouver and Lisbon. For example, one of us developed a youth-specific intake form for overdose prevention and safer injecting sites in Vancouver and has been active in advocating for a safe supply of drugs for YWPUD as part of the Vancouver Area Network of Drug Users (VANDU), Drug User Liberation Front (DULF), and Coalition of Peers Dismantling the Drug War (CPDDW)’s protests, during which tested, unadulterated heroin, meth, and cocaine were distributed to people who use drugs [13, 14, 18]. Others of us have long been advocating for the greater availability of harm reduction information and peer-led harm reduction services through networks such as Youth RISE and EuroNPUD. We have served as peer navigators for the first mobile drug consumption room that opened in Lisbon in 2019. One of us started the peer-led group MANAS in Lisbon, which includes young people. MANAS provides a space for women and non-binary people who use drugs and are experiencing violence and other intersecting vulnerabilities to come together and engage in artistic and wellness practices, provide mutual support, and access harm reduction supplies as well as sexual and reproductive health care, food, and clothing. It is informed by a commitment to fostering solidarity and engagement and recognizing intersectionality. The individuals involved in this group include those who are involved in sex work, living with HIV, and racialized, many of whom are navigating the institutional violence of child apprehensions. MANAS allows YPWUD to gather, imagine different futures together, and plan direct actions such as fighting for a permanent 24-h peer-run drop-in space.

## Young people use drugs

We know that young people—even very young people—use drugs. For example, many of us, as well as the people we have encountered through our research and life experiences, started using drugs (and not just cannabis) between the ages of 10 and 12. Young people use drugs for a lot of different reasons. Drugs are a source of sociality, pleasure, and fun, as well as a means of navigating boredom, physical, psychological and emotional pain, trauma, and forms of historical and structural oppression along axes of race, class, gender, sexuality, and ability [19, 20]. YPWUD in the context of street involvement often use stimulants such as meth and crack cocaine (crack) in order to stay awake outside for long periods of time to protect themselves and their belongings and to generate income [21]. Drugs themselves can be forms of harm reduction and treatment. For example, YPWUD in Vancouver and Lisbon use cannabis to mediate their use of substances such as crack, meth, heroin, and fentanyl [22]. In Vancouver, some YPWUD use meth to stop using crack, heroin, and fentanyl [21]. Stimulants like meth and crack can also be a means of mediating the sedating effects of methadone and some psychotropic medications. All of these examples demonstrate how YPWUD often carefully regulate their substance use to achieve desired effects and affects [20].

Young people use drugs, and they are also dying from overdoses. Since an official public health emergency was declared in the Canadian province of British Columbia (where Vancouver is located) in 2016, over 9230 people, including over 1720 young people under 30 years of age, have lost their lives to overdose [23]. Many of these deaths occurred among YPWUD in the context of street involvement. While Portugal is not in the midst of an overdose crisis, there are high and rising levels of heroin and crack use in this setting, and overdoses are increasing [24]. Young people use drugs for reasons that are often highly logical given their lived realities, and the need to reduce harms among YPWUD is clear. Despite this, harm reduction services, programs, and sites do not sufficiently meet the needs of youth in Vancouver and Portugal. When it comes to YPWUD, there continues to be an impetus towards the eradication of drug use. The goal is to “save” youth from drugs by encouraging them towards complete abstinence. In our experience, many parents, caregivers, providers, workers, and decision-makers seem to think that connecting young people with harm reduction will further “encourage” drug use rather than put a stop to it.

YPWUD often don’t get the chance to explain their drug use to the older people in their lives. They don’t get the chance to tell these individuals about what drugs are *doing* for them, such as helping to mediate physical, psychological and emotional pain and suicidality, stay safe and generate income, and find moments of pleasure and fun in the context of entrenched socio-economic marginalization, exclusion, and intimate and institutional violence. Instead, when a young person’s drug use is discovered in places such as schools, shelters, foster care and group homes, and certain types of government-subsidized housing environments, they are sometimes punished, including getting kicked out of these places [25, 26]. When YPWUD are not being driven away from care, they often face caregivers and providers who do not want to listen, and instead adopt a paternalistic “I know best” or “I need to save you” approach [26, 27]. BIPOC YPWUD in particular encounter paternalistic and judgemental attitudes in encounters with caregivers and providers, because systemic racism means that Black and Brown bodies and bodily practices are often viewed as inherently “at risk,” “risky,” and in need of intervention—oftentimes via hospitalization and incarceration [28].

In Vancouver and Lisbon, young people who use crack or meth on the streets are often framed as “aggressive,” “dangerous,” and “mentally ill,” and these understandings can combine with racism and other forms of structural oppression (classism, ableism, cis-heteropatriarchy) to reinforce paternalistic and coercive approaches to care on the part of caregivers, providers, and others [20]. In both settings, young people who use stimulants are frequently institutionalized in psychiatric units for treatment. While hospitalization may respond to YPWUD’s acute and short-term needs, detainment in psychiatric units can be a negative and traumatizing experience, particularly for YPWUD who have experienced multiple institutionalizations across their lives and across generations, as is the case for many Indigenous YPWUD in Canada [29]. Even so, there are growing calls across British Columbia for the involuntary hospitalization of YPWUD following overdose events [30].

## Care and control

Despite the relatively progressive policy landscapes of both Vancouver and Lisbon, the soft left hand of low-barrier harm reduction programs continues to be paired with the hard right hand of criminal sanctions and other forms of control in both settings [31]. In Vancouver and Lisbon, police are often tasked with identifying “problem” YPWUD and making referrals to services [11]. While accessing these services is technically voluntary in Portugal, physically presenting oneself before the Commissions for the Dissuasion of Drug Addiction is mandatory for those who are caught using drugs (including cannabis), and accepting “invitations to treatment” can be enforced by fines and other kinds of sanctions. In fact, the last decade has seen a sharp increase in criminal sanctions targeted at people who use drugs in Portugal, despite decriminalization [11].^4^ In Vancouver, people who use drugs in the context of street involvement continue to be heavily criminalized, and as mentioned above, there have been growing calls for the decriminalization of substance use in this setting [15].

Youth-dedicated drop-in centers and “one-stop-shop” service hubs that prioritize harm reduction are a better primary point of care for YPWUD than hospitals or criminal justice facilities. These kinds of centers and hubs do exist in Vancouver. They provide a range of harm reduction, drug use, mental health, and social services and are critical supports for YPWUD in this setting. In Portugal, harm reduction programs and centers are more explicitly targeted towards higher-income and older (> 18 years of age) YPWUD, such as those who use drugs at music festivals. In Lisbon, YPWUD in the context of street involvement have largely been left out of efforts to scale up harm reduction interventions, including in response to the COVID-19 pandemic [16].

In both Vancouver and Lisbon, YPWUD in the context of street involvement can experience challenges accessing centers, hubs, and programs even when they are included in program mandates. Many of these challenges arise from the everyday emergencies of ongoing poverty and addiction. Many YPWUD must daily navigate meeting their basic needs and high levels of violence connected to criminalized forms of income generation such as drug dealing and sex work. In this context, attending appointments or particular drop-in hours and modulating behavior to adhere to rules becomes difficult or impossible. Altercations with staff can lead to getting kicked out and avoiding these places in the future, cutting YPWUD off from much needed care and support. In Vancouver, some centers and hubs involve a level of surveillance that discourages YPWUD from accessing them. For example, in some places files are used to track how often a young person has accessed sterile drug use supplies, and this information may be shared with various providers and workers. Young people navigating pregnancy and parenting may be particularly afraid to go to these places out of a fear that if they access harm reduction supplies or seek help with their drug use, they may lose custody of children. Younger youth, and in particular younger Indigenous youth, may fear that accessing harm reduction supplies will precipitate a call to child protective services and removal from their families of origin, because systemic racism within the British Columbia child welfare system means that Indigenous children and youth are disproportionately placed in government care [25]. For YPWUD outside of major city centers, hubs and centers can be difficult to get to.

## Critical gaps

In Vancouver and Lisbon, there are other critical gaps in harm reduction services and programs for YPWUD as well. In both settings, youth-dedicated safer injection, safer smoking, and overdose prevention sites do not exist. The COVID-19 pandemic prompted a scaling up of harm reduction initiatives in both Vancouver and Lisbon [9, 16]. And yet, in Lisbon, interventions such as a new shelter that includes access to a safer consumption space (via a mobile drug consumption room) was not designed to include youth. In Vancouver, even when YPWUD are allowed to use those safer consumption sites that do exist, they often don’t feel comfortable in these adult-oriented spaces.

In our experience, adult-oriented safer consumption spaces can be intimidating for YPWUD, who don’t always feel like they can ask questions or get appropriate help in these places. In adult-oriented spaces, it can seem like everyone already knows what they are doing and what they *want* to be doing when it comes to their substance use, and many YPWUD feel like they have to imply that they are equally experienced and confident in their decisions about drugs when they are in these places. YPWUD may also worry that if they access adult-oriented safer consumption spaces, someone might report them to child protective services, or tell a family member, caregiver, provider, or worker that they were seen there. In Vancouver, we have seen YPWUD turned away from adult-oriented safer consumption spaces because they looked "too young" and “too healthy” to be using drugs intensively, or “didn’t have any track marks.” When YPWUD are uncomfortable or actively turned away, it can drive them even further away from life-saving care. It can also send the message that their lives are not worth saving.

In Portugal, safer drug consumption spaces in general are not widely available (the first safer smoking and injecting sites were opened in 2021), and there are no youth-dedicated spaces. Drug checking is only available in Lisbon, and take-home naloxone kits and peer-to-peer overdose prevention (naloxone) programs are also not available despite ongoing advocacy. In both Vancouver and Lisbon, there has been a primary focus on connecting people who use drugs with OAT and sterile drug use paraphernalia. As others have argued, the focus is on mediating drug-related risks and harms (e.g., syringe sharing, blood borne infections), and treating substance use “disorders” via licit replacement therapies (e.g., methadone, buprenorphine-naloxone), rather than on making the use of substances such as heroin, fentanyl, crack, and meth safer via safe supply and harm reduction programs [11]. In both settings, a focus on substance use as either criminal or pathological undermines the self-determination of YPWUD in relation to their drug use, harm reduction, and care.

## Nothing about us without us—including YPWUD

Harm reduction began as a grassroots initiative led by people who use drugs, activists, and their allies [2, 32]. In Vancouver and Lisbon, people with lived and living experience continue to rally behind the demand “Nothing about us without us,” which was first coined by disability rights activists in the 1990s [33] and became a mantra of the Vancouver Declaration manifesto released by the International Network of People Who Use Drugs [34]. And yet, in both Vancouver and Lisbon, YPWUD can be left out of grassroots harm reduction movements. For example, when the first drug users’ association was created in Portugal in 2009, there were no youth members. Today, youth can become members, and cooperation between older and younger people who use drugs is growing as activists push for the implementation of community-led harm reduction interventions (e.g., peer-run drop-in spaces) that have received greater attention for their efficacy during the COVID-19 pandemic [16].

In both Vancouver and Lisbon, we have observed that some of the benefits of harm reduction have been lost as the movement has become increasingly medicalized and professionalized. When harm reduction is spearheaded by clinicians, researchers, and other “professionals” rather than people with lived and living experience, it can become a part of the pathologization of drug use and people who use drugs. For example, in Vancouver it was once the norm to access harm reduction supplies and information from social spatial networks of people who use drugs, including YPWUD, engaged in “doing their own outreach out of their backpacks.” Today, many YPWUD access supplies from professionals they don’t know, sitting at the front desks of their housing buildings or clinics. These professionals can seem unfriendly and judgemental about handing out supplies to YPWUD and especially those under 18.

## Calls to action

To conclude, we make several calls to action to support the harm reduction needs of YPWUD in the context of street involvement in and beyond our settings:

1. We oppose approaches to preventing drug-related harms among young people, including very young people, that are premised on abstinence. We want accurate information about the risks and benefits of different drugs and how to practice different kinds of harm reduction in our schools and communities.
2. Young people’s engagement with harm reduction programs and sites should be kept confidential in order to encourage relationship- and trust-building and enduring connections with care. Those providing harm reduction to youth should always collaborate closely with them to determine what (if anything) regarding their drug use and harm reduction practices can be shared with family members, caregivers, staff, providers, and workers from the various systems of care and supervision that YPWUD often traverse.
3. We demand investment in low-barrier, youth-dedicated, and *youth-led* harm reduction programs and spaces, including safer consumption, drug checking, shelter, and housing sites. Ideally, youth-oriented safer consumption sites should have a non-clinical, relaxed feel to them, and include a welcoming drop-in space alongside private spaces for safer consumption. These should be places where YPWUD can access harm reduction supplies and information as well as food and other basic necessities. They should be staffed by a mix of peers and providers with experience providing non-judgemental care, support, and camaraderie to YPWUD. The focus should be on relationship-, trust-, and future-building, not damage, deficits, and the past [35].
4. Youth-oriented safer consumption, drug checking, shelter, and housing programs and spaces must account for the needs of youth who use stimulants and polysubstance-using youth. They must also account for the needs of BIPOC youth, gender diverse and queer youth, and self-identified young women. There should be dedicated programing and hours for young women (at least partially staffed by young women), gender diverse and queer youth (at least partially staffed by gender diverse and queer young people), and BIPOC youth (at least partially staffed by BIPOC young people). Mobile outreach vans and safer consumption rooms are critical to making harm reduction and other forms of support more accessible to YPWUD in the context of street involvement, and in particular those who are not residing in city centers.
5. Stop pathologizing YPWUD and trying to “save” or “fix” us. Recognize that we have the human right to make decisions for ourselves and keep ourselves safe. We demand an end to compulsory or involuntary abstinence-based treatment programs. Instead, we want to be listened to regarding what drugs *do* for us, socially, physically, mentally, and emotionally, in our daily lives [26]. Youth-dedicated drop-in centers and service hubs should center relationship- and trust-building and harm reduction, with treatments such as OAT easily available *to those who indicate they want these*. Comprehensive sexual and reproductive health services should also be available. When it comes to any kind of treatment, YPWUD should be empowered with decision-making power regarding plans and timelines [36]. In Portugal, we follow others in suggesting that appearance before the Commissions for the Dissuasion of Drug Addiction should be conditional on the consent of the young person, who should be able to decide whether to undergo any type of evaluation, diagnosis, and therapeutic intervention [11]. In Vancouver, we add our voices to those strongly cautioning against the involuntary detainment of YPWUD, including following an overdose event [29, 37, 38]. While we recognize that those providing care to YPWUD are often desperate to forcibly intervene, our research and experience demonstrates that involuntary approaches can push some youth further away from life-saving care, including during those moments when they are most vulnerable. For example, we know of instances when a young person hesitated to call emergency services while witnessing a friend overdosing, or attempted to escape from an ambulance, in order to avoid detention in hospital and other kinds of repercussions.
6. The services and systems that YPWUD traverse must be re-designed to foster youth’s self-determination in relation to their drug use, harm reduction, care, and families. YPWUD should be represented, engaged with, and empowered as citizens capable of making their own decisions. In our opinion, this is at the heart of what it means to practice trauma informed and culturally safe care. YPWUD who are in government care (i.e., living in foster care and group homes and independent living arrangements) must be able to access harm reduction services without fear of discipline and repercussions, including being placed in psychiatric wards, youth detention centers, and certain kinds of housing environments by social workers and teams as a “last resort” attempt to “fix” their substance use. These approaches are often isolating and traumatizing, leading young people to begin evading care altogether and putting them at greater risk for overdose and death. Youth who are aging out of government care and other services and systems must receive assistance with transitioning across services and systems, as well as continued financial support, so that they are not left without harm reduction and other kinds of support.
7. In Vancouver, we add our voices to those demanding the decriminalization of drug use, while at the same time cautioning that decriminalization—as it has played out in Portugal, for example—does not in and of itself constitute an end to the war on drugs [39]. We demand that YPWUD be at the table in a meaningful way (and not just as token participants in the process) as plans for decriminalization in Vancouver and shifts to the Portugal model are formulated and rolled out.
8. In Vancouver and Lisbon, we add our voices to those demanding a safe supply of drugs via peer-led compassion clubs that sell pharmaceutical grade cannabis, heroin, cocaine, and meth to those over 18 years of age [14].
9. Youth voices should be better integrated into both bottom up, grassroots *and* top down, state-sponsored harm reduction movements and initiatives. Harm reduction organizing and programming must be informed by an intersectional lens. It is critical to recognize and respond to how the risks and harms experienced by YPWUD are shaped by intersections of class, race, gender, sexuality, and ability. It is also important to recognize the intersections between drug user activism, anti-poverty activism, housing activism, and sexual health activism. Harm reduction organizing and action should make space for collective action and solidarity when there are overlapping positionalities, concerns, and priorities. It must also be cognizant of the need to hold space for those experiencing structural oppression (i.e., intersections of racism, classism, ableism, and cis-heteropatriarchy).
10. YPWUD in the context of greater privilege, as well as allies, should focus energy on fostering and extending the activism of YPWUD in the context of street involvement, creating vertical connections to power as well as horizontal connections across communities of people who use drugs. The goal is to grow a diverse and strong collective of YPWUD locally in Vancouver and Lisbon, as well as nationally and internationally.

## Data Availability

Not applicable.

## Footnotes

1 We acknowledge that this age range goes beyond many standard age range definitions of “youth” (e.g., “youth” as referring to those between 14 and 24 years of age). Nevertheless, we retain the use of the term, and use it somewhat interchangeably with “young people” throughout this piece, in order to signal how many of us view ourselves and our activism, even as we pass age 24. Many of us strongly assert our continued inclusion in the youth category, and view ourselves and our harm reduction needs as distinct from older populations of people who use drugs.

2 We recognize that the term “street involvement” is reductive and conceals as much as it reveals about the population that we are discussing in this comment. We use this term only for the sake of brevity (rather that repeating the descriptors of “homelessness, unstable housing, and ongoing poverty” throughout) and to draw attention to the more public nature of substance use among those who lack steady access to private, safe residences in which to consume drugs.

3 We were invited to author this comment by the journal as part of a special series on Young People, Drugs, and Harm Reduction. This special series is informed by an Advisory Board composed of young people who use drugs and their allies (also primarily young people under 30 years of age, as well as older PWUD and other experts). Feedback from this Advisory Board was used to generate a list of topics for invited comments, as well as a list of Advisory Board members who were interested in contributing to each. In composing the final author teams, we prioritized the inclusion of young people with lived and living experience in relation to the comment topics and also sought to bring young people together across geographies. DF coordinated the writing of the comment across several meetings with the author team, and also produced the first draft of the manuscript in collaboration with JC, KS, and KE. All other authors contributed to refining the content and writing across several meetings. Revisions to the comment were undertaken by DF, JC, and KS, with final approval being provided by all authors.

4 This shift is largely attributable to the 2008 Supreme Court Justice judgement (n. 8/2008) that re-established the crime of drug use (article 40, Decree-Law n. 15/93) when the quantity of drugs found on an individual exceeds the average individual use for a period of 10 days (behavior punishable by fines, community service, or even imprisonment for up to one year). Drug user activists in Vancouver have taken note of this shift in Portuguese drug policy and rising rates of criminal sanctions. As decriminalization is hotly debated in British Columbia and elsewhere across Canada, activists are demanding drug possession thresholds that will meaningfully curtail the police’s ability to criminalize people who use drugs.
